# Supplementary material for: Characterization of Global DNA Methylation in Different Gene Regions Reveals Candidate Biomarkers in Pigs with High and Low Levels of Boar Taint
Source: Vet Sci. 2020 Jun 13;7(2):77. doi: 10.3390/vetsci7020077 (PMC7356388; doi:10.3390/vetsci7020077)
Supplement: Supplementary file 1 [file vetsci-07-00077-s001.zip › vetsci-751747-supplementary_proofread_XW0611/Supplementary information_XW0611.docx]

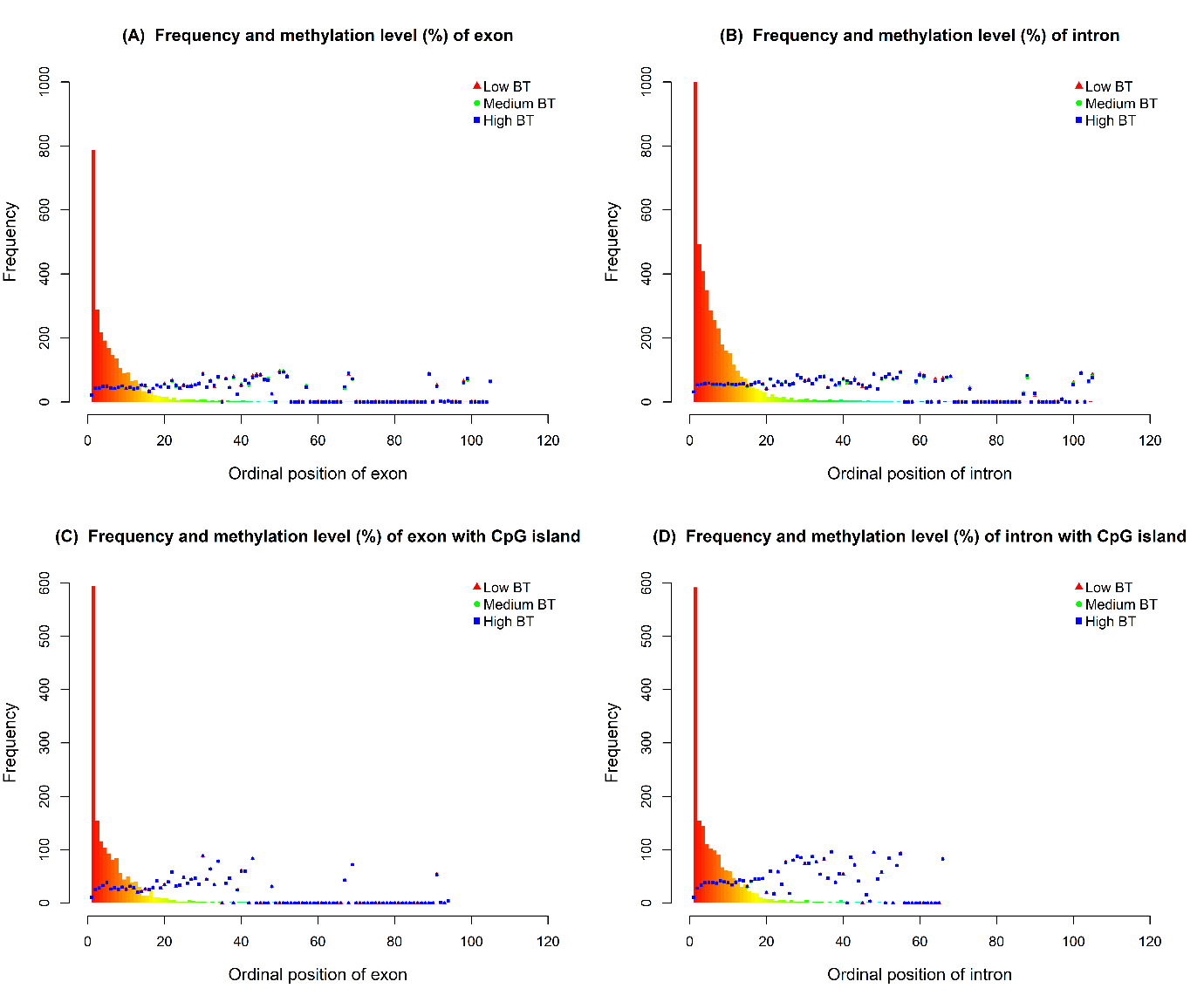


Supplementary Figure S1. Frequencies and methylation levels (%) for ordinal positions of (**A**) all exons, (**B**) all introns, (**C**) all exons with CpG islands and (**D**) all introns with CpG islands for low, medium and high BT groups. Note: Methylation level is zero when no cytosine is located in the ordinal position of the exon and intron.


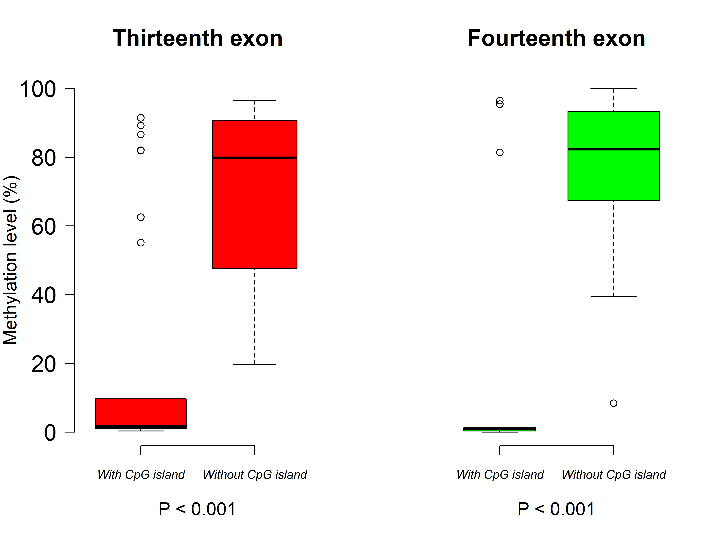


Supplementary Figure S2. Boxplots of methylation levels (%) of 13^th^ and 14^th^ exons with and without CpG island overlaps for low BT group.


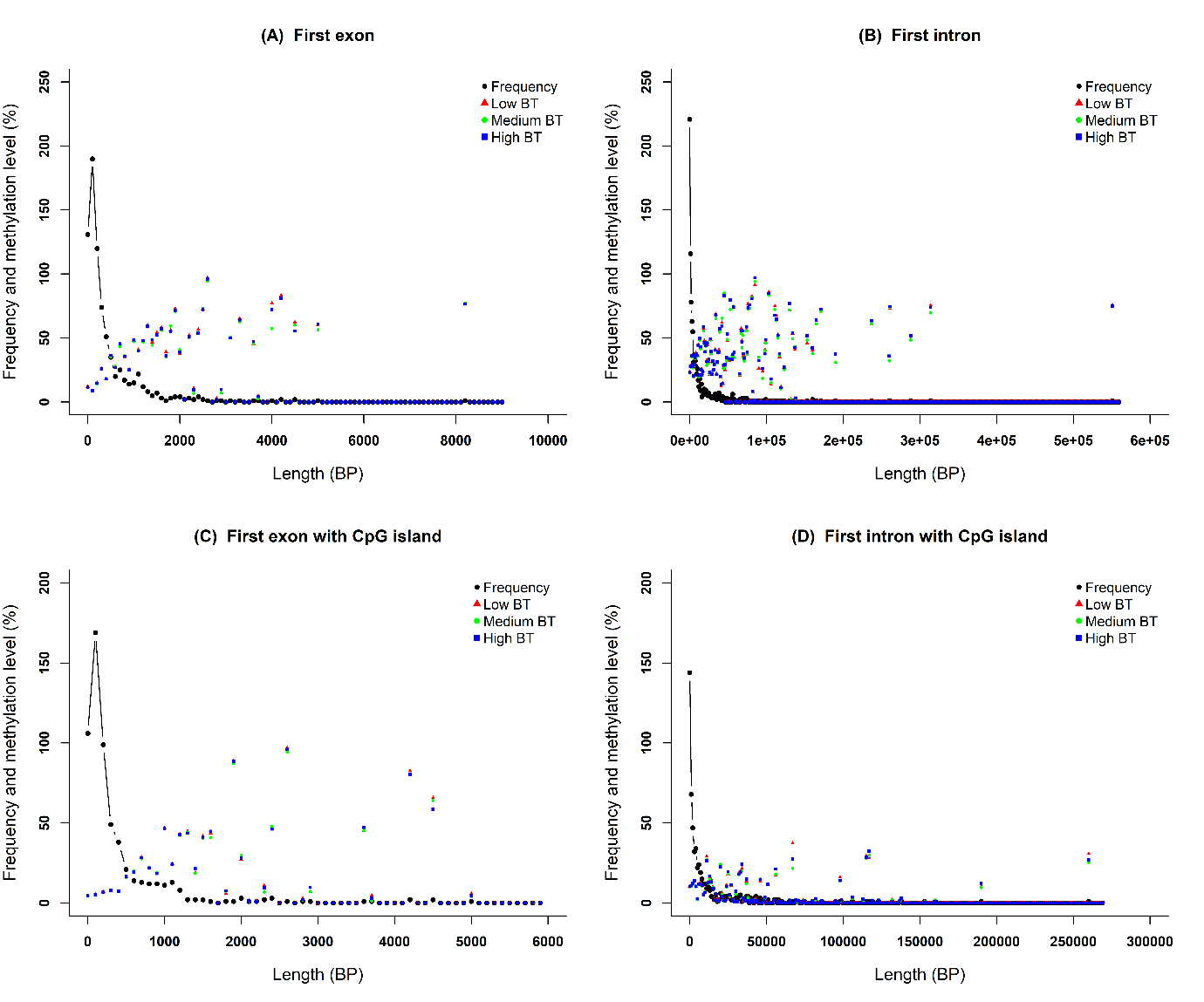


Supplementary Figure S3. Frequencies and methylation levels (%) for the (**A**) first exons, (**B**) first introns, (**C**) first exons of different lengths with CpG islands and (**D**) first introns with CpG islands in the low, medium and high BT groups. Note: The lengths of first exons and the first introns were categorized into 100 bp and 1,000 bp windows, respectively.


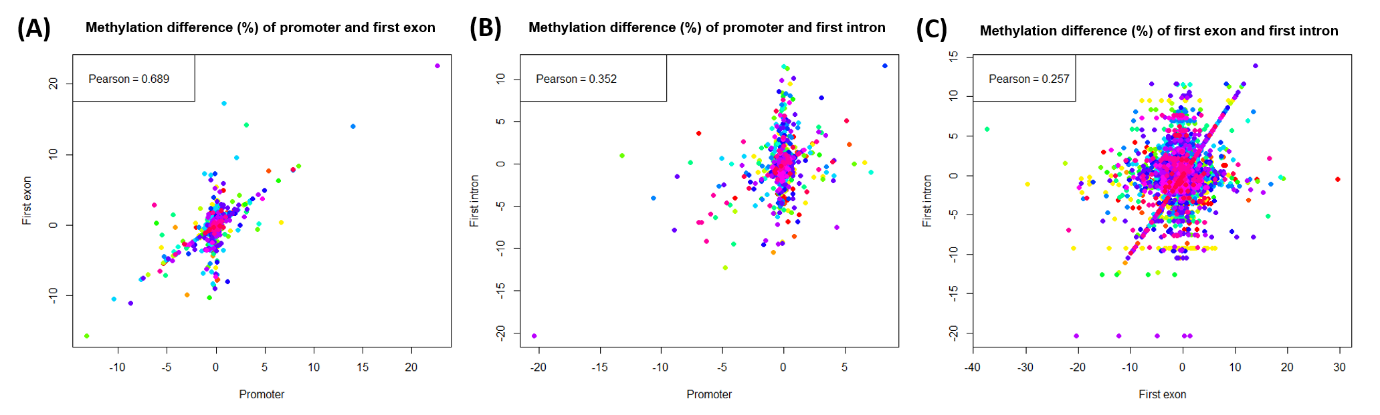


Supplementary Figure S4. Correlation plots of methylation differences (%) among promoters, first exons and first introns from the same genes. (**A**) Promoters and first exons. (**B**) Promoters and first introns. (**C**) First exons and first introns. Note: Methylation difference (%) refers to the difference in methylation levels between the low and high BT groups. Pearson refers to the Pearson correlation coefficient. The different colours of plots indicate the objects from different chromosomes.
